# Ethnopharmacological evaluation of schistosomicidal and cercaricidal activities of some selected medicinal plants from Ghana

**DOI:** 10.1186/s41182-020-00205-y

**Published:** 2020-04-10

**Authors:** Desmond Omane Acheampong, Ninette Owusu-Adzorah, Francis Ackah Armah, Enoch Aninagyei, Ernest Amponsah Asiamah, Ama Kyeraa Thomford, William Kofi Anyan

**Affiliations:** 1 Department of Biomedical Sciences, School of Allied Health Sciences, College of Health and Allied Science, University of Cape Coast, Cape Coast, Ghana; 2 Department of Biomedical Sciences, School of Basic and Biomedical Sciences, University of Health and Allied Sciences, Ho, Volta Region, Ghana; 3 Noguchi Memorial Institute for Medical Research, College of Health Sciences, University of Ghana, Legon, Ghana

**Keywords:** Cercariae, *Schistosoma mansoni*, *Azadirachta indica*, *Vernonia amygdalina*

## Abstract

**Background:**

The adulticidal and cercaricidal activities of five Ghanaian medicinal plants, namely, *Phyllanthus amarus*, *Vernonia amygdalina*, *Azadirachta indica*, *Morinda lucida* and *Nauclea latifolia* against *S. mansoni* were evaluated in this study. Six weeks old ICR mice (*n* = 25) were percutaneously infected with *S*. *mansoni* cercariae. Nine weeks later, infected mice (*n* = 5) were anaesthetised and perfused for adult *S*. *mansoni.* Cercariae were treated with different concentrations (1000, 500, 250, 125, 62.5, 31.25 μg/mL) of methanolic extracts of the experimenting plants in triplicates*.* Adult *S*. *mansoni* incopula were also treated with same concentrations of each extract or 20 μg/mL praziquantel. The cercariae and adult worms were observed at time intervals for 180 min and 120 h to assess mortality and viability respectively. Additionally, 9-week cercariae-infected mice (4 groups of 5 mice) were treated with either 500 mg/kg po *A. indica* or *V. amygdalina*, 400 mg/kg po praziquantel or distilled water for 14 days. The mice were euthanized after adult worms were recovered from them. The liver was processed and histologically examined for granuloma formations.

**Results:**

All the plants exhibited varying cercaricidal and adulticidal activities against *S. mansoni* in a time and concentration-dependent manner. *A. indica* (3 h IC_50_ = 27.62 μg/mL) and *V. amygdalina* (3 h IC_50_ = 35.84 μg/mL) exerted the highest cercaricidal activity. Worm recovery after treatment with *V. amygdalina*, *A. indica* and praziquantel in vivo was 48.8%, 85.1 % and 59.9 % respectively (*p* < 0.05). *A. indica* and *V. amydalina*-treated mice recorded lesser mean liver and spleen weights compared to untreated groups (*p* < 0.05).

**Conclusion:**

*A. indica* demonstrated the highest cercaricidal and alduticidal activities in vitro, whereas *V. amygdalina* exhibited the most potent aldulticidal activity in vivo. This study could provide baseline information which can be used to develop plant-based alternative commercial drugs against *S. mansoni*.

## Background

Schistosomiasis is a parasitic disease caused by the blood fluke *Schistosoma* and remains one of the important neglected tropical diseases globally. The average treatment cost per person per day is estimated to be $ 0.2–1.0 [1, 2]. The disease affects over 250 million people worldwide and causes, at least, 200,000 deaths annually [3]. Clinical manifestation includes, but not limited to, abdominal pain, hematuria, hematochezia and diarrhoea. In extreme cases, chronic schistosomiasis results in stunted growth and neurological disorders leading to learning difficulties, multi-organ damage, immuno-compromised system and, ultimately, death [4]. In *Schistosoma*-endemic countries, poverty is further deepened because the condition drastically reduces productivity. Currently, praziquantel (PZQ) is the drug of choice for treating human schistosomiasis [5]. Nevertheless, praziquantel has failed in many cases of juvenile worm (schistosomulae) infections because of drug insensitivity [6]. It is, therefore, imperative to find alternative therapies which are relatively affordable and readily available in the community.

A fundamental problem associated with the treatment of schistosomiasis and other infectious diseases is that, the pathogens mutate following selective pressure from the therapeutic agents. Additionally, in endemic countries, the disease is predominant in villages; treatment programmes, which are free, are mostly run in schools. Unfortunately, most of the children are seldomly in school, and some infected individuals are beyond school-going age. The infected individuals who are not covered by the treatment programme have to self-get the treatment regimen which, in most cases, is unaffordable to them [7, 8]. The aforementioned reasons necessitates the search for alternative therapies which are affordable and easily accessible to these category of people.

In Ghana, *Schistosoma haematobium* and *Schistosoma mansoni* are the main flukes that cause schistosomiasis [9]. The respective intermediate hosts, *Bulinus trancatus/globossus* and *Biomphalaria pfeifferi*, have been identified in several water bodies in Ghana [10]. Two stages in the life cycle of the parasitic helminth, miracidium and cercariae, are involved in the invasion of their hosts. Miracidium and cercariae respectively infect the intermediate host, snail, and definitive host, man [11]. Potential therapy targeted at any of the stages of the parasite’s life cycle could lead to treatment success.

Plants have played crucial roles in drug discovery and development by providing lead compounds for drug development [12]. Pathogens hardly develop resistance to their metabolites in most cases [13]. In view of these, plants and other natural products are mostly screened for metabolites with prophylactic and/or therapeutic potential against various conditions, including parasitic infections. For diseases of tropical origin, plant-based drugs come in handy partly; most tribes, due to have cultural connotations (folklore), perceive herbal medicine to be less toxic [14]. A large number of plant families with potential schistosomicidal activity have been identified through plant screening, which represents a continuous effort to find new bioactive molecules [15]. In Ghana, several plants (*Combretum mucronatum*, *Paullinia pinnata* and *Phyllanthus urinaria*) are used for treatment of helminth infections [16]; however, their cercaricidal and schistosomicidal properties have not been empirically assessed. This study, therefore, evaluated the cercaricidal and adulticidal activities of *Azadirachta indica*, *Morinda lucida*, *Nauclea latifolia*, *Phyllanthus amarus* and *Vernonia amygdalina* on *S. mansoni*.

## Results

### Cercaricidal activity of extracts

**Fig. 1:**
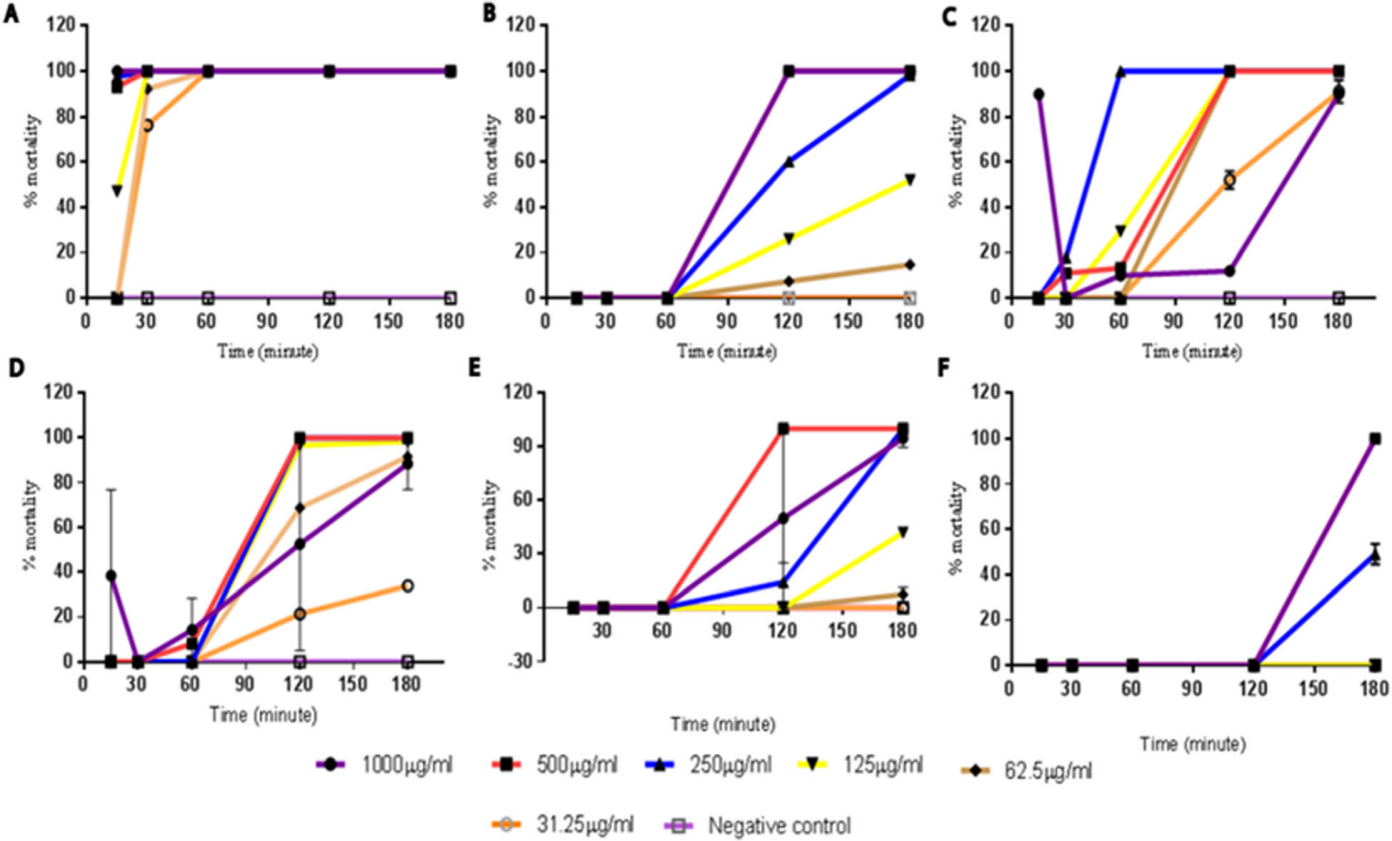
Time course mortality rate of the cercariae after treatment with the plant extracts. The percent mortality rate of cercariae after 180 min. **a***B. aegyptica*. **b***V. amygdalina*. **c***A. indica*. **d***N. latifolia*. **e***M. lucida*. **f***P. amarus*

Apart from *Balanites aegyptiaca* (positive control), the line graph of most of the extract concentrations remained steady at 0% mortality for 60 min or 120 min before they recorded a steady rise in percent mortality. The time-course graph for 1000 μg/mL *Nauclea latifolia* and *Azadirachta indica* rather followed an unusual pattern. The 1000 μg/mL extracts of *N. latifolia* and *A. indica* recorded a decline in mortality from 15 to 30 min before a steady rise. The test extracts achieved 100% mortality later than 30 min, in contrast to, *B. aegyptiaca* (125–1000 μg/mL), which produced 100% mortality from 15 to 30 min (Fig. 1a). Among the test extracts, only 250 μg/mL *A. indica* exhibited 100% mortality at 60 min; all other extract regimen achieved 100% mortality at 120 min. In Fig. 1, *B. aegyptiaca*, produced 100% mortality at 15 and 30 min at 125 μg/mL while *V. amygdalina* produced 98% mortality at 30 min with 250 μg/mL. Also, *A. indica* produced 100% mortality at concentrations from 250 μg/mL from 30 min and above while *N. latifolia* also produced 100% mortality at concentrations 250 μg/mL whereas *M. lucida* and *P. amarus* treatment had the highest onset of action with activity at 120th minute.

Additionally, *B. aegyptiaca* recorded the least IC_50_ values at all-time points. *A. indica* was next to record an IC_50_ value at 30 min and second smallest IC_50_ value for the remaining time points. *Vernonia amygdalina* was next with its first IC_50_ recorded at 60 min and third smallest IC_50_ value after *A. indica* and *B. aegyptica* for the remaining time points. *Morinda lucida* and *N. latifolia* were next to record their first IC_50_ values but at 120 min. However, *N. latifolia* recorded a smaller IC_50_ value than *M. lucida* and also for the remaining time points. *P. amarus* was the last to record an IC_50_ value at 180 min (Table 1). At the end of 180 min, the IC_50_ values from least to highest were 5.959 μg/mL (*Balanites aegyptiaca*), 27.62 μg/mL (*Azadirachta indica*), 35.84 μg/mL (*Vernonia amygdalina*), 117.7 μg/mL (*Nauclea latifolia*), 131.9 μg/mL (*Morinda lucida*) and 250.4 μg/mL (*Phyllanthus amarus)* (Table 1)*.*

**Table 1:** IC_50_ values of various extracts in μg/mL of the different time intervals

	IC_50_ Values of extracts (μg/mL)				
	15 min	30 min	60 min	120 min	180 min
*Balanites aegyptiaca*	127.5	18	5.959	5.959	5.959
*Azadirachta indica*	~	>1000	127.8	~ 31.22	27.62
*Morinda lucida*	~	~	~	262.3	131.9
*Nauclea latifolia*	~	~	~	195.9	117.7
*Phyllanthus amarus*	~	~	~	~	250.4
*Vernonia amygdalina*	~	~	>1000	48.1	35.84

### Adulticidal effect of plant extracts

#### Viability of adult schistosome worms in vitro

**Fig. 2:**
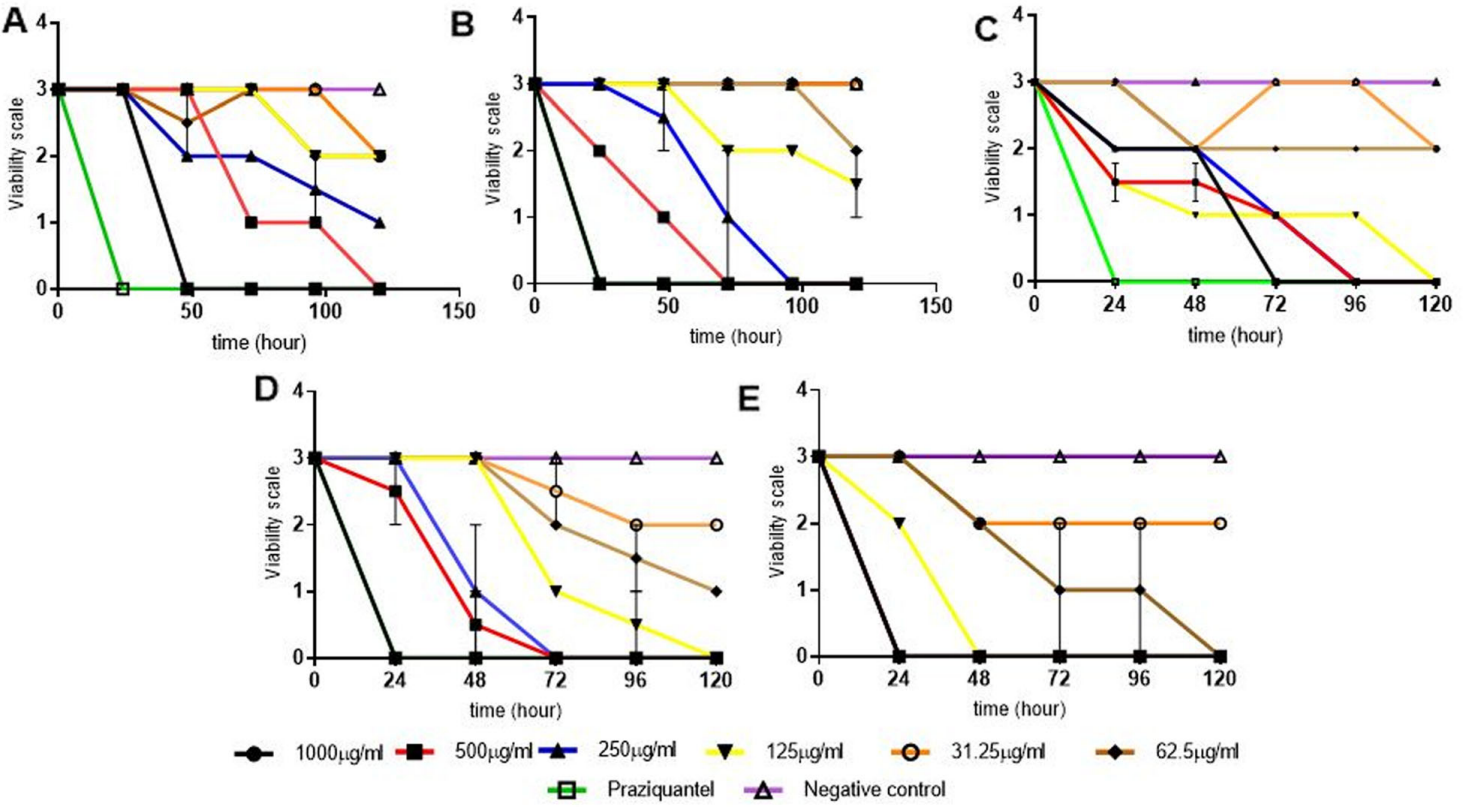
Anti-schistosomal activity of extracts. The percent mortality rate of the adult worm after *N. latifolia* (**a**), *V. amygdalina* (**b**), *M. lucida* (**c**), *P. amarus* (**d**) and *A. indica* (**e**) treatment. There was however no statistical difference between *A. indica* and PZQ

At the end of 120 h, most of the test extracts and praziquantel (positive control) were lethal to the adult incopula. The extract concentrations which reduced the viability scale from 3 to 0 include 62.5–1000 μg/mL *A. indica*, 125–1000 μg/mL *M. lucida* and *P. amarus*, 250–1000 μg/mL *V. amygdalina* and 500–1000 μg/mL *N. latifolia*. The negative control did not reduce the viability of the adult incopula throughout the experimental period (Fig. 2). It was noted that *N. latifolia* produced no worm viability after 48 h for any of the concentrations while *V. amygdalina* produced activity after 24 h, 48 h and 72 h for 1000 μg/mL, 500 μg/mL and 250 μg/mL respectively. Also *M. lucida* and *A. indica* produced no activity after 24 h for 24 h. Also *P. amarus* produced no mortality after 24 h for all the concentrations. There was however no statistical difference between *A. indica* and PZQ.

### Percentage worm recovery and worm burden

**Fig. 3:**
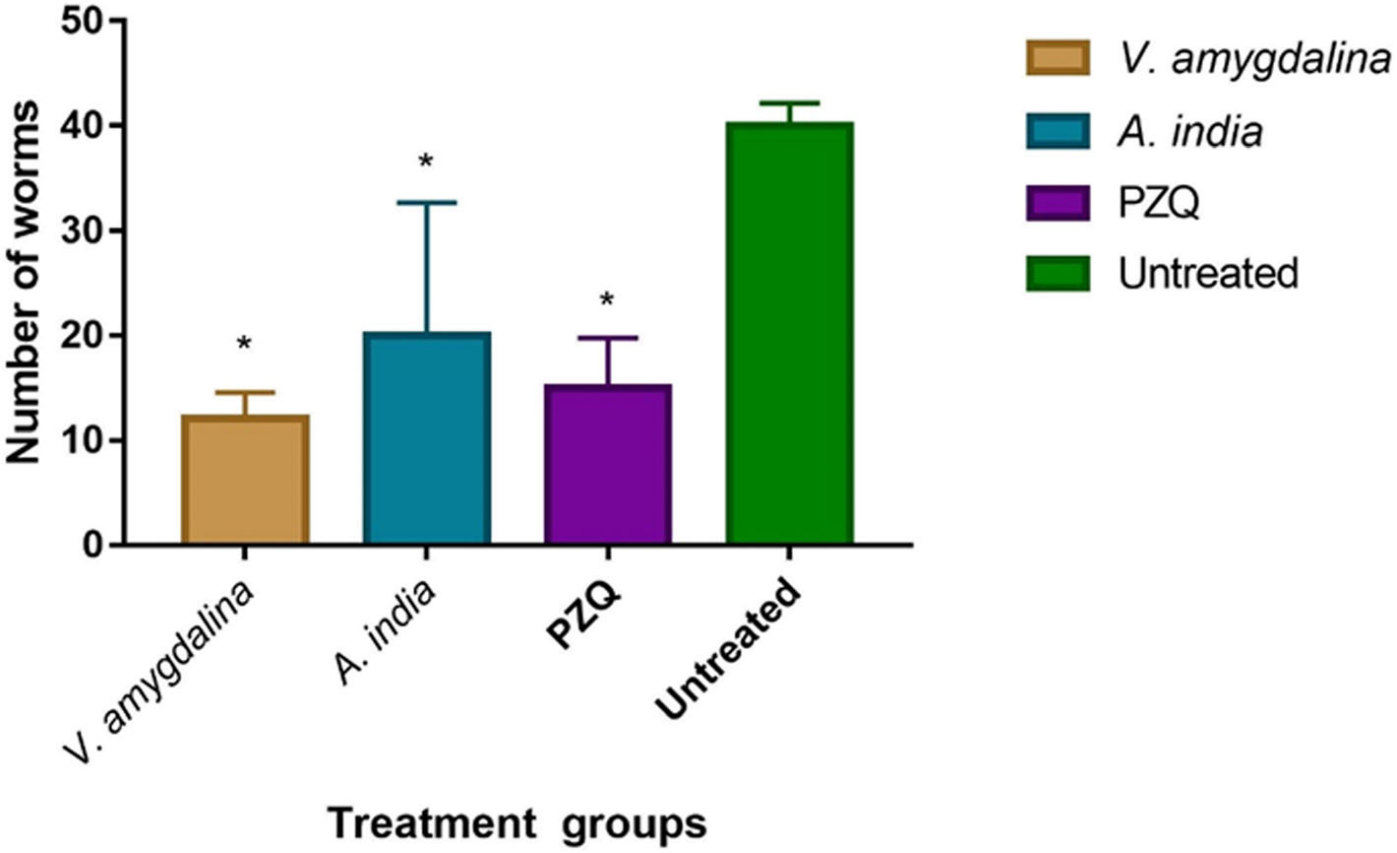
Mean worms recovered from treatment groups. The number of worms after treatment with the different extracts and PZQ. All treatment group compared with the untreated at *p* = 0.05 statistical significance

Further, *A. indica* and *V. amygdalina* plant extracts were selected for the *in vivo* studies based on their high in vitro schistosomicidal and cercaricidal activities. One-way analysis of variance (ANOVA) test showed that at least one of the mean numbers of adult worms recovered for a treatment group differed from another (*F* = 7.327, *p* = 0.0026). Post hoc analysis showed that the mean number of worms recovered from *V. amygdalina* (12.00 ± 1.549), *A. indica* (19.80 ± 8.194) and praziquantel (13.60 ± 3.600) -treated *S. mansoni* infected mice was significantly lesser than that of the untreated group (40.20 ± 3.072) (Table 2, Fig. 3).

**Table 2:** Worms recovered from *S. mansoni*-infected mice

Extract	Mean	F-ratio (*p*-value)
*V. amygdalina*	12.00 ± 1.549	
*A. indica*	19.80 ± 8.194	
Praziquantel	13.60 ± 3.600	7.327 (0.0026)
Untreated	40.20 ± 3.072

**Fig. 4:**
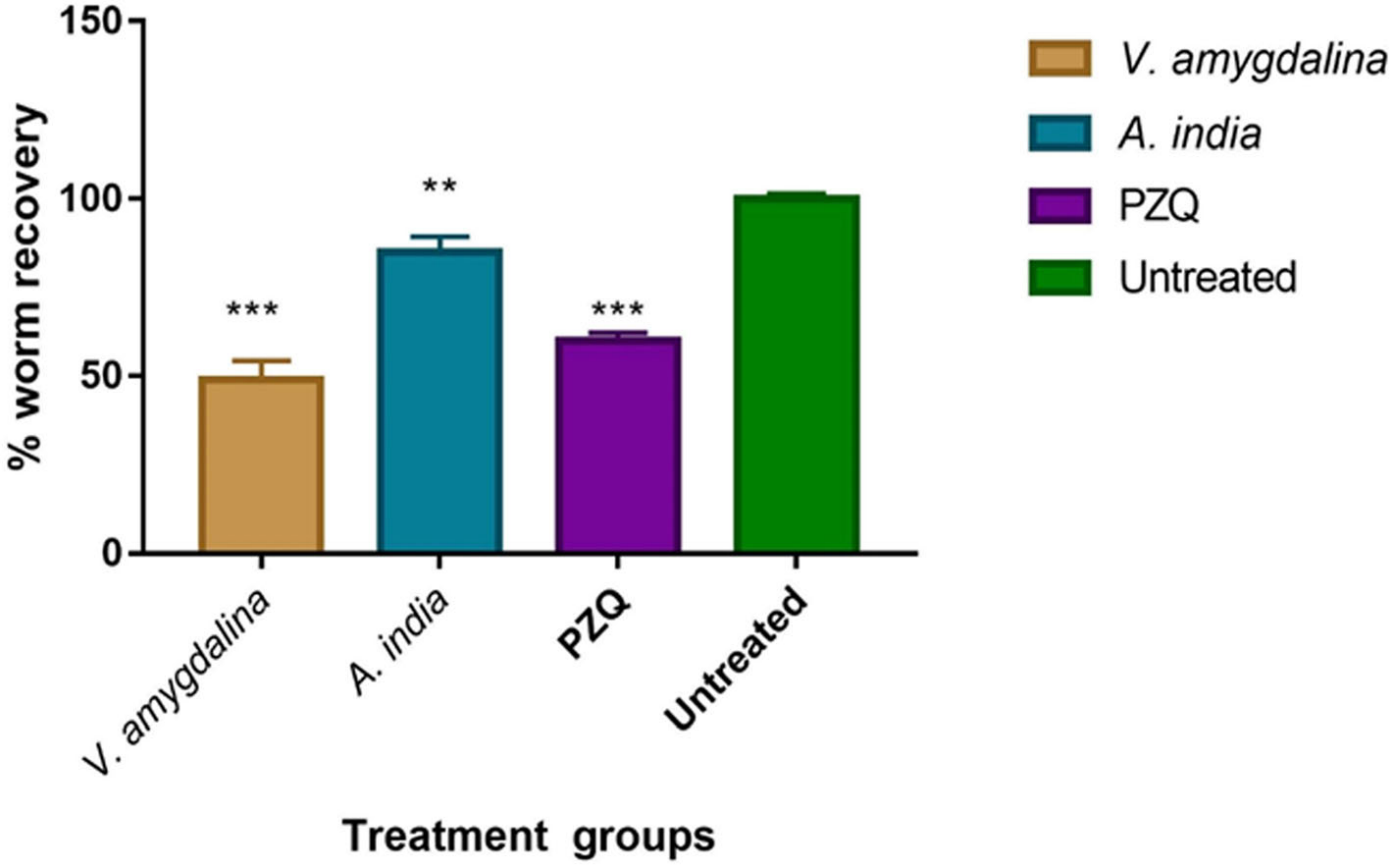
Percentage worm recovery

The mean percentage worm recovery values for the groups of mice treated with *V. amygdalina*, *A. indica* and praziquantel were 48.9%, 85.1% and 59.9 % respectively; they were significantly lesser than that of the untreated group (Fig. 4). However, there was no significant difference among the worm recovery values (*p* value = 0.669) of the treatment groups. The mean worm burden in the mice treated with *V. amygdalina* and praziquantel was 2, whereas, that of *A. indica*-treated and untreated mice scored a mean worm burden of 3. However, there was no significant difference in worm burden among the treatment options (*p* = 0.727). The average percentage worm recovery for the groups of mice treated with *V. amygdalina*, *A. indica* and praziquantel (positive control) was 48.9%, 85.1% and 59.9% respectively, relative to 100% recorded for the untreated mice (Fig. 4). However, there was no significant difference between the worm recovery following the treatment with *V. amygdalina*, *A. indica* and PZQ (*p* value = 0.669).

### Effect of extracts on the spleen and liver size and weight

**Fig. 5:**
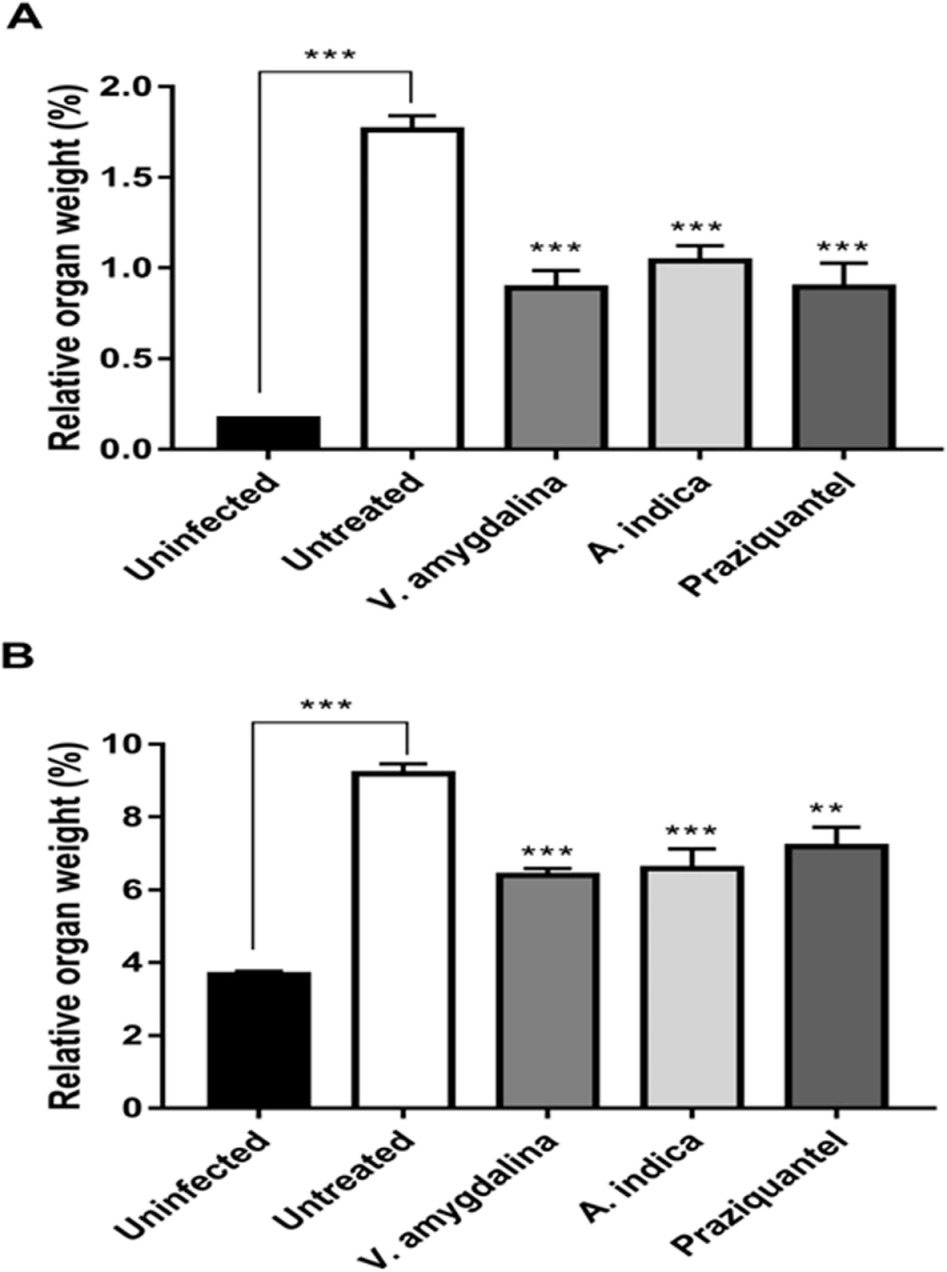
Relative organ weight (%) of the various treatment groups

The relative organ weights of the liver and spleen from *V. amygdalina*, *A. indica* and praziquantel (PZQ)-treated *S. mansoni* infected mice were all significantly lesser than that of the untreated group (*p* < 0.05) (Fig. 5a and b). The infected group recorded the least relative organ weight (3.8 g) and the untreated recording the highest (8.54 g). Treatment groups (*V. amygdalina* and *A. indica*) compared with the untreated group at *p* = 0.001 and PZQ compared with the untreated at *p* = 0.01 with a slim biological variation (indicated with error bars) in each case.

### Effect of extracts on granuloma formations in the liver

**Fig. 6:**
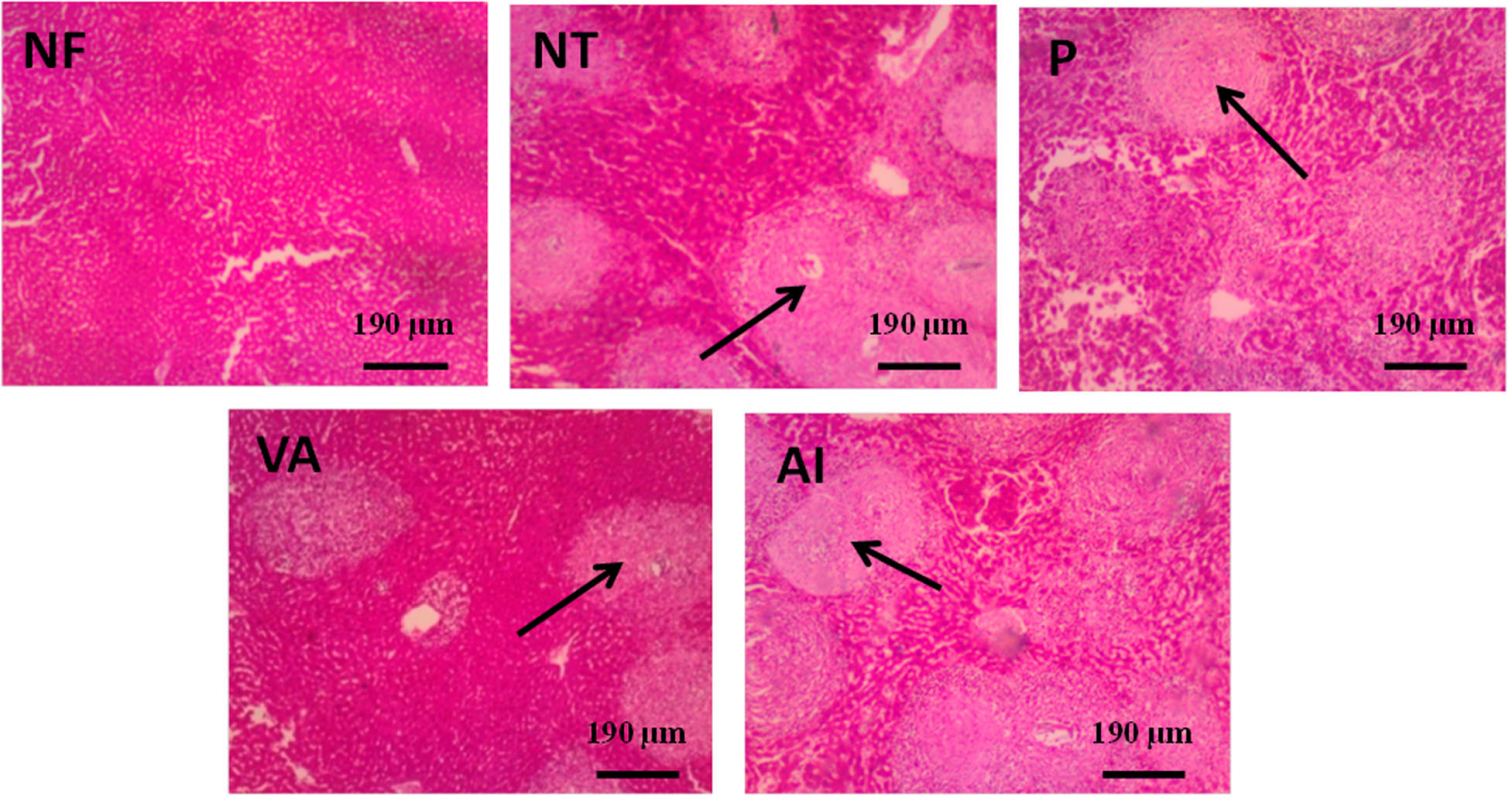
Photomicrographs (H&E × 200) of liver granuloma in *S. mansoni*-cercariae infected mice from various experimental groups. Single headed arrows () points to granulomas. NF—mice with no cercariae infection; NT—untreated cercariae infected mice; P—praziquantel-treated cercariae-infected mice; VA—*Vernonia amygdalina*-treated cercariae-infected mice; AI—*A. indica* treated cercariae-infected mice

The liver sections of the infected mice (both treated and non-treated groups) showed granulomas, which appeared as connective tissue fibres formed around an egg. The granuloma formations were severe (> 10) among all the untreated group. However, few to moderate granulomas (4 to 10) were observed in the treatment groups (Fig. 6). Granulomas were significantly smaller in diameter in *V. amydalina* and *A. indica* treatment groups than those in the untreated group (*p* < 0.05) (Table 3). Treated cercariae-infected mice group (P, VA and AI) had relatively less severe inflammatory cell infiltration compared with untreated NT group (Fig. 6).

**Table 3:** Mean diameter of granulomas from liver extract-treated cercariae-infected mice

Treatment	Mean diameter (μm)	95% CI diameter range of granulomas (μm)	*p* value	Post hoc analysis
Untreated	361.1 ± 25.02	279.5–392.7	< 0.0001	A–B 0.0907
				A–C 0.0001*
				A–D 0.0005*
PZQ	293.0 ± 10.90	268.4–317.7		B–C 0.0342*
				B–D 0.0828
*A. indica*	236.3 ± 7.36	219.9–252.8		C–D 0.6647
*V. amygdalina*	245.9 ± 9.45	223.9–266.6		

*C.I.* confidence interval

*indicates *p* < 0.05

## Discussion

In both tropical and sub-tropical regions, schistosomiasis is still a major public health concern. Schistosomiasis is prevalent in poor communities, where sanitation is poor and access to potable water is a challenge. Praziquantel is the drug of choice for treating all cases of schistosomiasis; however, cases of treatment failure have been reported in several studies due partly to resistance developed by the parasite towards praziquantel [17–21]. Considering the functional limitations associated with praziquantel, it has become imperative to investigate and discover alternative therapeutic agents with schistosomicidal activity, that are readily available, less expensive, biodegradable and less toxic [22].

This study, therefore, investigated and reports on both in vitro and in vivo effects of the methanolic extract of selected Ghanaian medicinal plants on different life stages of *S. mansoni*, the cercariae and adult worm. The methanolic extracts of the selected plants *A. indica*, *V. amygdalina*, *N. latifolia*, *P. amarus*, and *M. lucida* demonstrated cercaricidal and schistosomicidal potential in a dose- and time-dependent manner. *A. indica* exhibited the highest anti-cercarial activity among the plant extracts. Additionally, *A. indica* exhibited the highest in vitro activity against the adult incopula, comparable to that of standard drug praziquantel. Thus, *A. indica*, upon further investigation, could be developed into an alternative agent for the treatment of schistosomiasis. These results corroborate other studies elsewhere [23, 24] and thus, demonstrate that *A. indica* could play an important role in any biological control program aimed at irradicating schistosomiasis. However, at low dose (< 500 μg/mL), dose response was not observed for *N. latifolia*, *M. lucida* and *P. amarus*. This observation could be as a result of tolerance of these plants at low doses. This could be possible as cercaria may have been exposed to low doses of these plants in their natural environment.

Statistically, *V. amydalina* has comparable adulticidal activity as *A. indica* in vitro*.* In contrast, the former registered a relatively higher anti-schistosomal index in vivo. Interestingly, *V. amygdalina* exerted a better adulticidal activity than the standard drug praziquantel in vivo, similar to results reported by Adediran and Uwalaka [25]. Their study proves that *V. amygdalina* is relatively more potent against helminths than conventional drugs ivermectin, levamisole and albendazole, attesting to its efficacy. Additionally, a study by Ogboli et al. [26] also reports that the leaf extracts of *V. amygdalina* have curative effects on mice infected with *Schistosoma mansoni* [25–28]*.* Undoubtedly, *V. amygdalina* demonstrates that it possesses anti-schistosomal activity and should, therefore, be investigated further to derive the full benefit. In this study, the efficacy of *A. indica* and *V. amygdalina* was studied in the same experiment unlike the previous studies. Moreover, these plants were collected from different geographical locations. Again, the efficacy of these plants were compared to others plants whose anti-schistosomal efficacy was not known.

The pairing of the male and female worms in the human host brings about sexual maturation and production of eggs. Moreover, most of the pathologies in schistosomiasis are as a result of the schistosome eggs [28]. The host immune responses towards the eggs and its antigens result into granuloma formation in the liver and hepatosplenomegaly in schistosomiasis patients [29]. In this study, the spleen and liver of the praziquantel-treated cercariae-infected mice was relatively larger compared to those treated with *V. amygdalina*, suggesting an attenuated hepatomegaly and splenomegaly in the latter group. Furthermore, more granulomas were observed in the hepatic parenchyma of praziquantel-treated mice as opposed to the *V. amygdalina* treated group, further suggesting *V. amygdalina* as better alternative to praziquantel. The acute and sub-acute toxicities of *A. indica* and *V. amygdalina* have been previously reported and were found to be safe [30, 31]. These plants ability to reduce the size of granulomas is a testament to its efficacy.

Additionally, we are of the view that the activity of *V. amygdalina* is possibly exerted through disruption of adult worm pairing and/or inhibition of egg production in the female worm; hence, few granulomas formed. This could, possibly, be the mechanism of action of *V. amygdalina*; however, more robust molecular techniques have to be employed to establish its mechanism of action.

## Conclusion

All of the five plant extracts, *P. amarus*, *M. lucida*, *N. latifolia*, *V. amygdalina*, and *A. indica* exerted varying levels of cercaricidal and adulticidal activities against *S. mansoni*. However, whereas *A. indica* exerted the highest cercaricidal and adulticidal activities in vitro, *V. amygdalina* exhibited the highest schistosomicidal activity in vivo. More importantly, *V. amygdalina* and *A. indica* demonstrated higher activity in vivo and in vitro relative to the respective standard drugs. Hence, *V. amygdalina* and *A. indica* are potential agents for the management of schistosomiasis. Based on the therapeutic potential exhibited by these plant extracts, further studies will be necessary to elucidate the specific compounds responsible for the antischistosomal activities and establish the molecular mechanisms involved. Knowledge of these will facilitate their development into therapeutic agents.

## Methods

### Preparation and extraction process of plant specimens

The whole plant of *P. amarus*, leaves of *V. amygdalina* and *A. indica*, and both the leaves and bark of *M. lucida* and *N. latifolia* were collected from the Botanical Gardens of the University of Cape Coast in September 2017. The plant specimens were identified and authenticated at the Herbarium Unit, School of Biological Sciences, University of Cape Coast, Ghana. A voucher specimen *P. amarus* (5152), *V. amygdalina* (4521), *A. indica* (3717), *M. lucida* (3193) and *N. latifolia* (6623) were kept at the Biological Sciences Herbarium, University of Cape Coast for reference. Dried *B. aegyptiaca* seeds were obtained from the Herbarium Unit, School of Biological Sciences, University of Cape Coast, Ghana. *B. aegyptiaca* was used as a positive control due to its known schistosomicidal activity [32, 33] while *A. indica* and *V. amygdalina* have variable anti-schistosomal activity, *N. latifolia*, *P. amarus*, and *M. lucida* were selected based on their anti-microbial activities in previous studies and their abundance in Ghana.

The extraction was performed adopting a previously described methodology [34] but with few modifications. Fresh parts of each plant specimen were washed and air dried at 25 ^°^C for 7 days and subsequently in an oven at 40 ^°^C for 20 min. The dried plant materials were ground into powder. Exactly 400 g of each pulverized plant material was cold macerated with 800 mL of absolute methanol at 25 to 30 ^°^C for 48 h. The mixture was filtered, and the filtrate concentrated using rotary evaporator (Buchi Rotavapor, R 200) under reduced pressure at 40 ^°^C. The crude extract was finally dried in a desiccator and percentage yield of the plant extracts calculated, as shown in supplementary table 1 (table S1).

### Experimental animals: maintenance and infection

#### Maintenance and infection of intermediate snail host, shedding and estimation of cercaria

Matured *B. pfeifferi* (shell diameter 10–12 mm) snails were maintained in the snail laboratory at the Noguchi Memorial Institute for Medical Research (NMIMR), Accra, Ghana under standard laboratory conditions of 6.6–8.4 pH, 18–28 °C and 7–9 dGH (70–90 ppm of calcium). The snails were each exposed to 4–6 miracidia for 5 weeks. Ten infected snails, observed under light microscope to be shedding cercariae, were then transferred into a test tube containing enough distilled water to submerge them. The snails in the test tube were exposed to artificial light for 120 min to stimulate cercariae shedding. The cercariae suspension was subsequently drawn into a new test tube and at three different times, 50 μL of the cercariae suspension was transferred onto a microscope slide to estimate the number of cercariae per millilitre, using light microscope aided by a tally counter [35]. The cercariae suspension was used for in vitro cercaricidal activity and infection of mice for in vivo adulticidal activity.

#### Maintenance and infection of mice with cercariae

Twenty-five 6-week old female ICR mice were purchased from the Animal Experimental Unit of the Centre for Research into Plant Medicine (CRPM), Mampong Akuapem, Ghana. The mice were allowed to acclimatize at the animal housing facility of the School of Biological Sciences, University of Cape Coast for 1 week under the following conditions: ~ 18–23 °C temperature, 40–60% relative humidity and 12 h light/dark cycle. The mice were fed on standard pellet diet (Grower Mash, Essaar, Ghana) and provided water ad libitum. Twenty-five (25) of the mice were infected percutaneously as follows. Each mouse was placed in a glass restrainer and its tail then placed in an approximately *n* = 150 *S. mansoni* cercariae suspension for 60 min. The mice were handled in accordance with the standard guidelines as enshrined in the “Principles of laboratory animal care” (NIH publication No.85-23, revised 1985) as well as the specific national and institutional requirements regarding the use of animals in scientific studies.

#### In vitro cercaricidal activity of the plant extracts

Serial dilutions (1000 μg/mL, 500 μg/mL, 250 μg/mL, 125 μg/mL, 62.5 μg/mL and 31.25 μg/mL) of each of the test plant extracts were prepared using distilled water as diluent. Two millimetre of each dilution was added to three wells of a 6-well culture plate. Approximately thirty cercariae were then pipetted into each of the wells. The wells were observed using an inverted light microscope at specific time intervals: 15, 30, 60, 90, 120, 150 and 180 min. The number of dead cercariae at various time points was counted. *Balanites aegyptiaca* was used as positive control and RPMI +1% dimethyl sulfoxide (DMSO) as the negative control.

#### In vitro evaluation of the anti-schistosomal activity

In vitro adulticidal activity was carried out as described previously [36] with few modifications. Five 8-week old *S. mansoni* cercariae-infected mice were randomly selected, euthanized with intraperitoneal injections of pentobarbital/heparin solution. Perfusate citrate saline (0.85% sodium chloride; 1.5% sodium citrate) was pumped into the descending thoracic aorta using a 20 gauge needle. A slit was made in the hepatic portal vein and the perfusate was collected into a container for adult worms’ recovery. The recovered worms were washed thrice with phosphate-buffered saline (PBS) (pH = 7.4) and finally in RPMI 1640 medium. Subsequently, incopula adult worms were picked into each well of a 24-well culture plate containing RPMI 1640 medium supplemented with 1% HEPES, 10% fetal bovine serum (FBS) and 100 μg/mL penicillin. A 2 mL of 1000 μg/mL, 500 μg/mL, 250 μg/mL, 125 μg/mL, 62.5 μg/mL and 31.25 μg/mL of the plant extracts were added to the well in triplicates, each containing a pair of the worms. Some of the paired worm-wells were treated with either 20 μg/mL praziquantel or RPMI 1640 medium with supplements +1% DMSO to serve as positive and negative controls, respectively. The plates were then incubated at 37 °C with 5% CO_2_ for 120 h and observed under an inverted microscope at 24-h interval. The medium was also changed every 48 h to ensure that there was sufficient nutrient for the worms. Viability of the worms per well was scored as follows: 0—worm dead; 1—severe reduction in motility and morphological changes; 2—reduced motility and first morphological changes; and 3—vital, normally active and no morphological changes.

#### In vivo treatment with plant extracts and granuloma size determination

Nine-week *S. mansoni* cercariae-infected mice were randomly put into four groups of five: infected mice + *A. indica* (500 mg/kg po), infected mice + *V. amygdalina* (500 mg/kg po), infected mice + praziquantel (400 mg/kg po) and infected mice + distilled water (infected control). The two plant extracts, *A. indica* and *V. amygdalina* were selected based on their better activity against the adult worms in vitro*.* Two weeks after treatment, the mice were anaesthetised with sodium citrate and perfused with normal saline for adult worm recovery and counting with the aid of an inverted microscope (Olympus CK 300). The percentage worm recovery was calculated as follows: % worm recovery = mean of total worms in experimental group/mean of total worms in infected control × 100%. Also, total number of paired adult worms per mouse was recorded as the worm burden. The liver and spleen were isolated after which, the weight and organ to body weight of the various treatment groups were determined.

#### Histopathological examination of the liver tissues

The right lobe of the liver from each mouse was fixed in 10% buffered formalin and taken through routine histological processes previously described by Gyasi et al. [37]. After trimming of tissue block, random sections (5.0-μm thickness) were stained with haematoxylin-eosin (HE). With the aid of the light microscope (Olympus CX41RF) coupled to a digital camera (Olympus soft imaging solutions GMBH), granulomas, which presented as fibrous connective tissue surrounding an ovum were identified and counted. The cross-sectional areas of the granulomas were determined using an integrated measurement tool of the LMscope software (Micro-Tech Lab, Austria). The granulomas in the livers were grouped as none (0), few (1 to 3), moderate (4 to 10) and severe (> 10) granulomas per liver with reference to their mean diameters [38].

### Statistical analysis

All data were expressed as mean ± standard deviation (SD). A time-course percent mortality and time-course viability scale line graphs were created for in vitro anticercarial and antischistosomal activities respectively. GraphPad Prism for Windows version 4.03 (GraphPad Software, San Diego, CA, USA) was used for all statistical analyses. p<0.05 was considered statistically significant.

## Supplementary information

**Additional file 1.** Supplementary Tables and Figures.

## Data Availability

All data generated or analyzed during this study are included in this published article.

## Abbreviations

ANOVA: Analysis of variance
DMSO: Dimethyl sulfoxide
FBS: Fetal bovine serum
HE: Hematoxylin eosin
NMIMR: Noguchi Memorial Institute of Medical research
NTDs: Neglected tropical diseases
PZQ: Prazequantel
