# Supplementary material for: Ethnopharmacological evaluation of schistosomicidal and cercaricidal activities of some selected medicinal plants from Ghana
Source: Trop Med Health. 2020 Apr 10;48:19. doi: 10.1186/s41182-020-00205-y (PMC7147056; doi:10.1186/s41182-020-00205-y)
Supplement: Supplementary file 1 — Additional file 1. Supplementary Tables and Figures. [file 41182_2020_205_MOESM1_ESM.pdf]

Supplementary Tables and Figures

Table S1: Percentage yield of plant extracts

Plant	% yield
M. lucida (leaves and bark)	2.03
P. amarus (whole plant)	5.38
V. amygdalina (leaves)	9.61
N. latifolia (leaves and bark)	3.11
A. indica (leaves)	1.56

Table **S2** : Histopathology of treated *S. mansoni* infected mice

Treatment group	Inflammation	Granulomas
V. amygdalina (V) 500mg/kg		
V1	√ S	MG
V2	√ S	MG
V3	√ S	FG
V4	√ S	FG
V5	√S	MG
A. indica (A) 500mg/kg		
A1	√ S	MG
A2	√ S	MG
A3	√ S	FG
A4	√ S	NG
A5	√S	MG
Praziquantel (P) 400mg/kg		

P1		√ S	MG
P2		√ S	MG
P3		√ S	MG
P4	√*		
P5	√*		
Untreated (NT)			
NT1		√ *	SG
NT2		√*	SG
NT3		√*	SG
NT4		√*	SG
NT5		√*	SG

√-pathology, S–slightly inflamed, *-very inflamed,

0: none granuloma (NG); 1–3: few granulomas (FG); 4–10: moderate granulomas (MG); > 10: severe granulomas (SG)

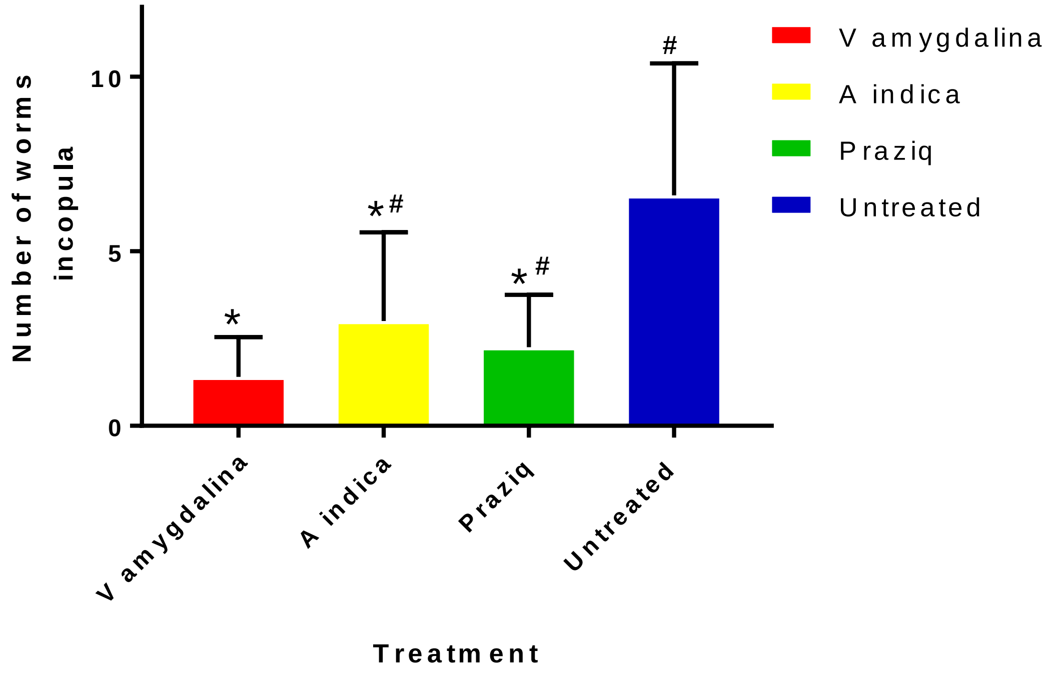

Figure S1: Worm burden of *S. mansoni* infected mice
